# LEARNING FROM THE IMPLEMENTATION OF INTEGRATED COMMUNITY-BASED PROGRAMS FOR OLDER ADULTS WITH HIGHER NEEDS

**DOI:** 10.1093/geroni/igad104.2862

**Published:** 2023-12-21

**Authors:** Bobbi Symes, Kahir Lalji

**Affiliations:** United Way British Columbia, Burnaby, British Columbia, Canada; United Way British Columbia, Burnaby, British Columbia, Canada

## Abstract

Three demonstration projects of integrated community-based programs for older adults with higher needs were implemented in British Columbia over 2020 to 2023: 1) Social Prescribing (SP, n=19 programs), 2) Therapeutic Activation Programs for Seniors (TAPS, n=15 programs), and 3) Family and Friend Caregiver Groups (FFCG, n=16 programs). This presentation reports on findings on the programs’ design and implementation based on data collected via interviews and surveys of program staff. Since inception, the total number of unique participants programs have served is 1,078 for TAPS, 1,900 for FFCG, and 1,397 for SP. The majority of clients have been long-term clients receiving services for 3 or more months. Due to the COVID-19 pandemic, 80% of programs reported they had shifted their focus during their first year of operations in response to emergent pandemic needs, thus impacting their start-up and initial intake of clients. The flexibility provided to programs to pivot to offering needed supports during the height of the pandemic was considered an important success. Across all types of programs, client who were socially isolated or lacked social supports were perceived as one of the groups who benefited the most from the programs. Programs reported that their relationships with community-based organizations, primary care networks, and home and community care were strengthened and over three-quarters had formed at least three new partnerships. A variety of facilitating (e.g., leveraging pre-existing relationships) and limiting factors (e.g., awareness of programs and obtaining referrals) were identified that have affected the success of program implementation.

